# Diagnosing Beyond Bias: Differentiating Topical Steroid Withdrawal Syndrome From irAE-Induced Adrenal Insufficiency

**DOI:** 10.7759/cureus.50272

**Published:** 2023-12-10

**Authors:** Toshio Suzuki

**Affiliations:** 1 Department of Medical Oncology, University of Tsukuba, Tsukuba, JPN

**Keywords:** diagnostic error, steroid ointment, oral mucositis, topical steroid withdrawal, immune-related adverse event, iatrogenic adrenal insufficiency

## Abstract

Confirmation bias can impede accurate diagnosis. Since the approval of immune checkpoint inhibitors (ICIs), physicians are on high alert for newly developed immune-related adverse events (irAEs). Therefore, when patients present irAE-like symptoms, there is a risk of confirmation bias leading to overlooked diagnoses. This paper discusses a case of a patient with non-small cell lung cancer treated with nivolumab and topical dexamethasone ointment for an extended period due to oral mucocutaneous irAE. The patient developed adrenal insufficiency, initially considered to be a likely case of pituitary irAE. However, further investigation and the patient's clinical course revealed an unexpected diagnosis more in line with topical steroid withdrawal syndrome.

## Introduction

Immune checkpoint inhibitors (ICIs) have revolutionized cancer treatment, leading to an increase in long-term cancer survivors. While ICIs are effective, they can also induce a range of immune-related adverse events (irAEs) [1,2]. Cutaneous irAEs, including lichenoid reactions, eczema, vitiligo, and pruritus, are particularly common [3,4]. These reactions, recently reported in oral mucosa as well, are typically self-limiting and manageable with topical steroids, allowing for the continued use of ICIs [1,5]. The phenomenon of steroid addiction, known in patients on long-term systemic corticosteroids, is less understood in the context of topical steroid use, particularly in the oral mucosa. This paper reports a lung cancer patient who developed secondary adrenal insufficiency after the inadvertent discontinuation of dexamethasone ointment, which was prescribed for nivolumab-induced oral mucositis. Initially, the clinical presentation was suggestive of a pituitary irAE during nivolumab therapy. However, careful reassessment and the patient's clinical course pointed towards a diagnosis more aligned with topical steroid withdrawal syndrome.

## Case presentation

Three years prior, a 73-year-old male was diagnosed with a postoperative recurrence of left lung adenocarcinoma (pathological T2aN0M0 (pT2aN2M0), American Joint Committee on Cancer (AJCC) Stage IIIA) and had been under chemotherapeutic management. He presented to the clinic with severe appetite loss and dehydration following the sixteenth cycle of third-line treatment with nivolumab. Upon admission, physical examination revealed an Eastern Cooperative Oncology Group (ECOG) performance status (PS) of 4, body temperature of 37.2℃, blood pressure of 102/51 mmHg, heart rate of 128 bpm, and a respiratory rate of 20/min. His oral mucosa was notably dry, but there was no evidence of thyromegaly, heart murmurs, chest rales, rash, or skin pigmentation. He reported experiencing diarrhea for three days one week prior to admission, followed by subsequent appetite loss and anorexia. However, CT on admission showed no signs of active colitis, other sources of infection, or tumor progression.

Laboratory tests indicated metabolic acidosis, renal failure, a low serum sodium level, and an elevated eosinophil count, but normal thyroid hormone and glucose levels (Table 1). Hormonal examination revealed a markedly low level of early morning plasma adrenocorticotropic hormone (ACTH) and a mildly decreased serum cortisol level. Other pituitary hormones, including prolactin (PRL), luteinizing hormone (LH), and follicle-stimulating hormone (FSH), were elevated (Table 1). MRI of the pituitary gland and hypothalamus did not reveal any abnormalities, such as enlargement or space-occupying lesions.

**Table 1:** Laboratory data on admission Hgb: hemoglobin; UN: urea nitrogen; Cre: creatinine; Na: sodium; FPG: fasting plasma glucose; TSH: thyroid stimulating hormone; FT3: free triiodothyronine; FT4: free thyroxine; ACTH: adrenocorticotropic hormone; PRL: prolactin; LH: luteinizing hormone; FSH: follicle stimulating hormone; AVP: arginine vasopressin

Labs	Results	Units	Reference range
WBC	11,900	/μl	4,000-9,000
Neutrophil	45.9	%	45-55
Lymphocyte	15.3	%	25-45
Monocyte	7.7	%	4-7
Eosinophil	29.3	%	1-5
Basophil	1.8	%	0-1
Hgb	16.9	g/dl	14-18
UN	27.2	mg/dl	8-20
Cre	1.8	mg/dl	0.61-1.04
Na	132	mEq/l	135-147
FPG	94	mg/dl	70-199
TSH	2.06	μIU/ml	0.5-5
FT3	1.8	pg/ml	2.3-4
FT4	1.27	ng/dl	0.9-1.7
ACTH	<1.5	pg/ml	7.2-63.3
Cortisol	6.3	μg/dl	6.4-21
PRL	89.1	ng/ml	3.6-10.3
LH	35.2	mIU/ml	0.79-5.72
FSH	72.6	mIU/ml	1.27-19.26
AVP	2.7	pg/ml	<4.0

Based on these findings, suppression of the hypothalamic-pituitary-adrenal (HPA) axis was definitively diagnosed. Initially, the etiology was presumed to be a pituitary irAE, as he had been suffering from an oral lichenoid reaction, another irAE, for the past year. Concurrently, our differential diagnosis included iatrogenic Cushing’s syndrome due to the long-term use of dexamethasone oral ointment (0.1%, approximately 2g daily) for irAE-associated mucositis, and a subsequent steroid withdrawal syndrome following his recent illness. A detailed interview revealed that he had discontinued the dexamethasone ointment after the onset of diarrhea and decreased oral intake.

IV hydrocortisone (100 mg bolus) was immediately administered, followed by oral hydrocortisone (30 mg daily). He recovered within two days post-admission, and the hydrocortisone dosage was gradually reduced to 15 mg daily. On day 14 post-admission, a rapid ACTH stimulation test (250 μg) was performed to assess for primary and secondary adrenal insufficiency. The test, conducted 24 hours after discontinuing hydrocortisone and dexamethasone ointment, revealed a basal cortisol level of 1.2 μg/dL, with levels remaining low (4.7 μg/dL and 6.3 μg/dL at 30 and 60 minutes post-ACTH administration, respectively). A retrospective CT comparison showed adrenal gland atrophy at the time of admission. Despite initial consideration of irAE-induced adrenal insufficiency due to the patient’s history of oral mucosal irAE and current presentation, the clinical course and additional findings gradually pointed toward a more likely diagnosis of topical steroid withdrawal syndrome, involving suppression of the HPA axis.

Following the discontinuation of oral hydrocortisone and ongoing use of dexamethasone oral ointment, no significant symptom recurrence was noted one-month post-cessation. Notably, daily variability in dexamethasone ointment application led to intermittent symptoms indicative of adrenal insufficiency. To address this, a maintenance small dose of hydrocortisone at 10mg/day was reintroduced, effectively stabilizing symptom fluctuations attributable to inconsistent ointment dosing. Additionally, guidance was provided to prevent excessive use of the dexamethasone oral ointment, leading to sustained symptom stability over the long term.

## Discussion

In this case, where the patient had previously developed irAE affecting the oral mucosa, the emergence of adrenal insufficiency symptoms led to the initial suspicion of a pituitary irAE in the differential diagnosis. However, a meticulous and comprehensive assessment later revealed that the cessation of steroid oral ointment was more plausibly the cause, manifesting as a withdrawal syndrome. While it is impossible to differentiate between the two with absolute certainty, this delineation offers a profound educational insight, underscoring the importance of thorough evaluation in complex clinical scenarios encountered in oncologic practice. This differential diagnosis is particularly challenging due to the overlapping clinical presentations of irAEs and steroid withdrawal syndrome, a distinction that holds significant implications for patient management.

Unlike most reported cases of adrenal insufficiency associated with ICIs, which are typically attributed to direct immune-mediated damage to the adrenal or pituitary glands [6], our case was unique due to the involvement of topical steroid withdrawal. This distinction is crucial, as misattributing adrenal insufficiency to irAEs could lead to unnecessary immunosuppressive therapy, potentially exacerbating the underlying condition [7].

Furthermore, the phenomenon of topical steroid withdrawal, especially in the context of oral mucosal application, is less documented in current literature [8-10]. However, in cases where patients are using large amounts of topical steroids, physicians must always be cognizant of the risk of steroid withdrawal syndrome. It is likely that only a few physicians are fully aware of the actual amount of topical steroids used. In this case, it became apparent that the patient had been applying approximately 1g of dexamethasone daily to the oral mucosa. After his condition improved, it was coincidentally discovered that on days when the patient inadvertently omitted both oral steroids and topical applications, symptoms similar to those at admission reappeared, strongly suggesting that the clinical course was attributable to steroid withdrawal syndrome.

The case also underscores the importance of a thorough and unbiased evaluation in patients presenting with adrenal insufficiency while on ICI therapy, a stance supported by recent studies emphasizing the diverse and often unpredictable nature of irAEs [11,12]. These findings highlight the need for increased vigilance and a broader differential diagnosis when managing similar cases in the future.

The incidence of irAEs is frequently unpredictable, necessitating vigilant monitoring for their emergence in patients treated with immune checkpoint inhibitors. This heightened awareness, however, can predispose clinicians to a confirmation bias when symptoms mimicking irAEs present, potentially leading to an inadvertent marginalization of alternative differential diagnoses. Such a bias might inadvertently emphasize irAEs, at times overshadowing the critical consideration of other pertinent medical conditions.

## Conclusions

In summary, this case underscores the diagnostic complexities in patients with adrenal insufficiency following ICI therapy. It highlights the need for vigilance toward differential diagnoses like pituitary irAE and topical steroid withdrawal syndrome. Physicians must maintain an open mind and avoid confirmation bias, especially when managing patients with complex presentations.
